# Cancer Stem Cells and the Renin–Angiotensin System in the Tumor Microenvironment of Melanoma: Implications on Current Therapies

**DOI:** 10.3390/ijms26031389

**Published:** 2025-02-06

**Authors:** Ethan J. Kilmister, Swee T. Tan

**Affiliations:** 1 Gillies McIndoe Research Institute, Wellington 6242, New Zealand; 2 Wellington Regional Plastic, Maxillofacial and Burns Unit, Hutt Hospital, Lower Hutt 5010, New Zealand; 3 Department of Surgery, The University of Melbourne, Royal Melbourne Hospital, Parkville, VIC 3052, Australia

**Keywords:** melanoma progression, melanoma metastasis molecular background, renin–angiotensin system, acquired drug resistance, drug repurposing, immunotherapy, targeted therapy, therapeutic targets, cancer stem cells

## Abstract

Multiple signaling pathways are dysregulated in melanoma, notably the Ras/RAF/MAPK/ERK and PI3K/AKT/mTOR pathways, which can be targeted therapeutically. The high immunogenicity of melanoma has been exploited using checkpoint inhibitors. Whilst targeted therapies and immune checkpoint inhibitors have improved the survival of patients with advanced melanoma, treatment resistance, their side effect profiles, and the prohibitive cost remain a challenge, and the survival outcomes remain suboptimal. Treatment resistance has been attributed to the presence of cancer stem cells (CSCs), a small subpopulation of pluripotent, highly tumorigenic cells proposed to drive cancer progression, recurrence, metastasis, and treatment resistance. CSCs reside within the tumor microenvironment (TME) regulated by the immune system, and the paracrine renin–angiotensin system, which is expressed in many cancer types, including melanoma. This narrative review discusses the role of CSCs and the paracrine renin–angiotensin system in the melanoma TME, and its implications on the current treatment of advanced melanoma with targeted therapy and immune checkpoint blockers. It also highlights the regulation of the Ras/RAF/MAPK/ERK and PI3K/AKT/mTOR pathways by the renin–angiotensin system via pro-renin receptors, and how this may relate to CSCs and treatment resistance, underscoring the potential for improving the efficacy of targeted therapy and immunotherapy by concurrently modulating the renin–angiotensin system.

## 1. Introduction

Melanoma is the most aggressive form of skin cancer, accounting for 90% of all deaths from skin cancer [1]. Its incidence continues to rise globally, particularly among fair-skinned populations and individuals over 60 years of age [2]. The etiopathogenesis of melanoma involves complex interactions between environmental, genetic, and epigenetic factors [1].

**Figure 1:**
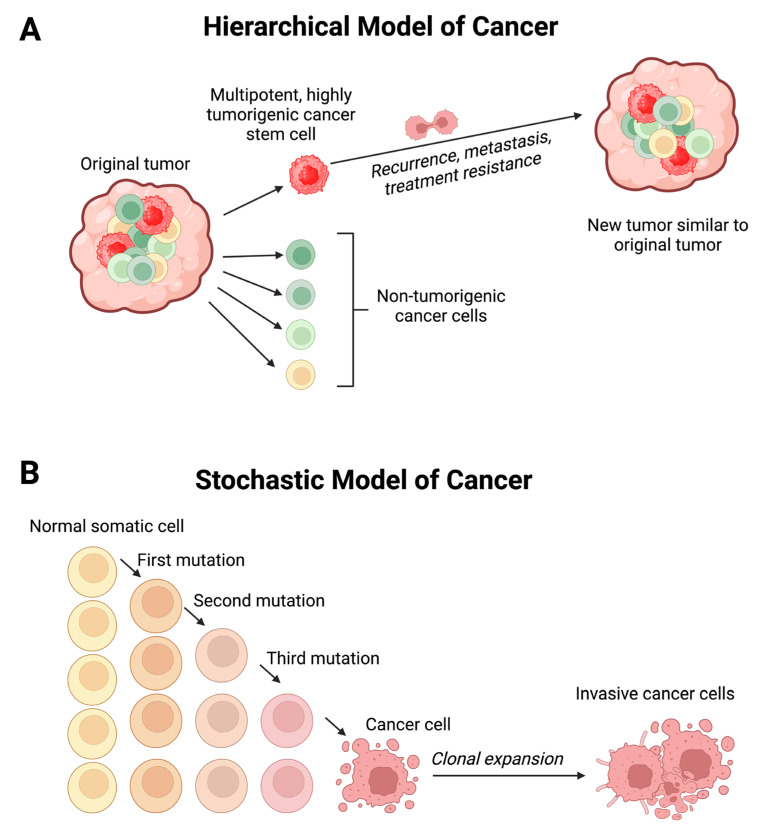
(**A**) The hierarchical model of cancer proposes the presence of a highly tumorigenic cancer stem cell (CSC) sitting atop the tumor cellular hierarchy, giving rise to identical CSCs and differentiated cancer cells through asymmetric cell division, resulting in heterogenous tumor cell populations. (**B**) The stochastic model of cancer proposes that a normal somatic cell accumulates oncogenic mutations in a stepwise manner and becomes a cancer cell that undergoes clonal expansion to form a tumor. Figure modified and reproduced with permission from *Biomedicines* [13].

Melanoma can metastasize to any organ at varying frequencies. An autopsy study of 216 cases revealed metastases in lymph nodes (73.6%), lungs (71.3%), liver (58.3%), brain (49.1%), bone (48.6%), heart (47.2%), adrenal glands (46.8%), and gastrointestinal tract (43.5%) [3]. Poor outcomes for melanoma have been attributed to the presence of cancer stem cells (CSCs) in both primary [4] and metastatic [5,6,7] melanoma. CSCs exhibit pluripotency and self-renewal capabilities, collectively termed stemness, generating identical CSCs and differentiated cancer cells through asymmetric cell division. This process creates heterogenous tumor cell populations, as proposed by the hierarchical model of cancer [8] (Figure 1A). Cancer stemness—defined as the degree to which cancer cells display stem cell properties, including self-renewal, proliferation, and multilineage differentiation—is often used interchangeably with CSCs [9]. CSCs are attributed to cancer recurrence, metastasis, and resistance to various therapeutic modalities, including chemotherapy, radiotherapy [10], and immunotherapy [11], ultimately leading to treatment failure [8]. In contrast to the hierarchical model of cancer, the stochastic model (also known as the clonal evolutional theory) of cancer proposes that tumors arise from the stepwise accumulation of genetic and epigenetic alterations in a normal somatic cell, conferring heritable survival advantages to this cancer cell, which undergoes clonal expansion to form a tumor [12] (Figure 1B).

The pathogenesis of melanoma involves multiple dysregulated signaling pathways. Mutations in *BRAF*, *NRAS, NF1,* and *KIT* drive overactivation of the Ras/RAF/MAPK/ERK and PI3K/AKT/mTOR signaling pathways, promoting melanoma progression [14]. While targeted inhibitors such as dabrafenib (a BRAF inhibitor) and trametinib (a MEK inhibitor) improve the survival outcomes of patients with *BRAF V600E* or *V600K* mutations [15,16], drug resistance typically emerges within months due to the activation of alternative pathways involved in cellular proliferation, angiogenesis, and metastasis [17].

The high immunogenicity of melanoma [18] has led to various therapeutic approaches that manipulate the immune system. While earlier attempts with melanoma vaccines showed limited efficacy [19], recent developments of mRNA vaccines, particularly those tested in the mRNA-4157-P201KEYNOTE-942 phase II clinical trial, show promising therapeutic potential [20].

Advances in immunotherapy, especially checkpoint inhibitors, represent a significant breakthrough in the treatment of metastatic melanoma. Ipilimumab, a cytotoxic T-lymphocyte-associated protein 4 (CTLA-4) blocking antibody, approved by the FDA in 2011, demonstrates prolonged survival in patients with metastatic melanoma [21]. Subsequently, programmed cell death protein 1 (PD-1) inhibitors (nivolumab and pembrolizumab) show superior survival outcomes compared to ipilimumab and conventional chemotherapy [22,23,24]. The EORTC1225-KEYNOTE-054 trial shows improved recurrence-free survival and distant metastasis-free survival in patients with resected stage III melanoma [25,26], with survival benefits extending to completely resected stage IIB or IIC melanoma [27]. Recent trials, such as the I/II RELATIVITY-048 trial investigating immune-oncology triplet therapy (ipilimumab, nivolimumab, and relatilimab), show an objective response rate of 58.7% and a 48-month overall survival rate of 69.1%, with manageable safety profiles [28]. mRNA-4157 (V940), an individualized mRNA vaccine, in combination with pembrolizumab, reduces the risk of recurrence or death by 44% in patients with resected high-risk stages IIIB/C/D and IV melanoma (HR = 0.561; 95% CI 0.30 to 1.017) [20]. However, drug resistance, adverse effects, and the prohibitive cost of these novel therapies necessitate continued exploration of more effective, better tolerated, and more affordable treatment options.

The renin–angiotensin system, traditionally known as an endocrine system for its role in cardiovascular homeostasis [29], is increasingly appreciated to play a key role in regulating CSCs within the tumor microenvironment (TME) in a paracrine fashion (paracrine renin–angiotensin system) [13,30,31]. The TME comprises multiple components, including blood vessels, extracellular matrix, signaling molecules, soluble molecules, inflammatory and immune cells, fibroblasts, keratinocytes, smooth muscle cells, and endothelial cells [32,33]. Interactions between melanoma CSCs and the TME, influenced by the paracrine renin–angiotensin system [31,33], is suggested by the expression of components of the renin–angiotensin system by phenotypic CSCs in metastatic melanoma [6,34]. We have identified components of the renin–angiotensin system in metastatic melanoma to the head and neck lymph nodes [34] and the brain [35]. However, there are limited studies investigating the renin–angiotensin system in other specific metastatic melanoma subsites. This article explores the interplay between CSCs and the paracrine renin–angiotensin system in the melanoma TME, and their interaction with the dysregulated Ras/RAF/MAPK/ERK and PI3K/AKT/mTOR signaling pathways, highlighting their implications on current therapeutic approaches.

## 2. Dysregulated Signaling Pathways in Melanoma

Multiple dysregulated signaling pathways in melanoma, including the Ras/RAF/MAPK/ERK and PI3K/AKT/mTOR pathways, drive tumor progression. PI3K, AKT, and mTOR have been shown to be overactive across various cancer types, including melanoma [36]. These components are also essential in embryogenesis, with their absence often resulting in embryonic developmental failure [37,38,39]. Multiple studies have demonstrated that PI3K/AKT/mTOR signaling preserves the ability of pluripotent stem cells to self-renew and differentiate. Human embryonic stem cells (ESCs) require PI3K/AKT signaling to maintain an undifferentiated state, while the inhibition of PI3K leads to the loss of pluripotency [40]. Inhibition of the PI3K/AKT/mTOR pathway may affect the differentiation and behavior of resident CSCs [34]. This is relevant as pro-renin receptor (PRR) is expressed on the phenotypic CSCs that express SOX2, a marker employed to identify CSC, in metastatic melanoma [34].

Overactivation of the Ras/RAF/MAPK/ERK pathway occurs in 98% of melanoma [41], predominantly due to mutations in the *BRAFV600* and *Ras* genes [42]. MEK inhibitors (trametibinib, cobimetinib, and binimetinib) have been used in combination with BRAF inhibitors to address MAPK-driven treatment resistance, with 15–20% of melanoma patients with the *BRAFV600E* mutation not responding, which has been attributed to the loss of the tumor suppressor genes *PTEN* and *NF1* [42].

Resistance to targeted inhibitors develops through several mechanisms, most commonly through reactivation of the Ras/RAF/MAPK/ERK pathway, as observed in 80% of BRAF inhibitor-resistant tumors, resulting from BRAF protein alterations through overexpression, alternative splicing, and heterogeneity in *BRAFV600* [43]. Additionally, resistance mechanisms include the PI3K/AKT/mTOR pathway, which is dysregulated in 22% of BRAF inhibitor-resistant melanomas characterized by increased expression of the AKT protein [36]. This dysregulation stems from mutations in *AKT1* and *AKT3*, or *PIK3CA* and *PTEN* with a loss of function. Epidermal growth factor receptor signaling, leading to pathway reactivation, is another contributor to resistance to targeted inhibitors [42].

In this article, we explore the mechanisms of resistance to targeted inhibitors, focusing on the interaction between the paracrine renin–angiotensin system within the melanoma TME and the Ras/RAF/MAPK/ERK and PI3K/AKT/mTOR pathways, as well as the role of CSCs regulated by these pathways. We explore the potential impact of these interactions on resistance to immunotherapy and targeted therapies.

## 3. The Role of the Paracrine Renin–Angiotensin System in Melanoma

### 3.1. The Renin–Angiotensin System

**Figure 2:**
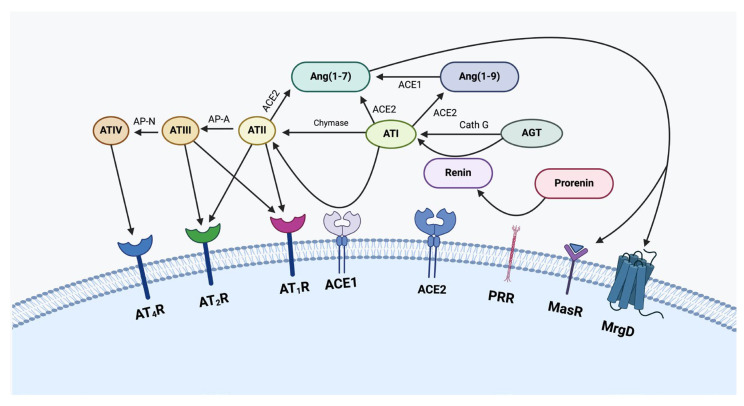
A schema showing the paracrine renin–angiotensin system (see text). PRR, pro-renin receptor; Cath G, cathepsin G; Cath B, cathepsin B; Cath D, cathepsin D; ACE1, angiotensin-converting enzyme 1; ACE2, angiotensin-converting enzyme 2; AGT, angiotensinogen; Ang(1–7), angiotensin (1–7); Ang(1–9), angiotensin (1–9); AP-A, aminopeptidase-A; AP-N, aminopeptidase-N; ATI, angiotensin I; ATII, angiotensin II; ATIII, angiotensin III; ATIV, angiotensin IV; AT_1_R, angiotensin II receptor 1; AT_2_R, angiotensin II receptor 2; AT_4_R, angiotensin II receptor 4; MrgD, Mas-related-G protein coupled receptor; MasR, Mas receptor. Reproduced and adapted with permission from *Biomedicines* [13].

The classical endocrine renin–angiotensin system cascade (Figure 2) physiologically begins with angiotensinogen (AGT), which is synthesized and secreted by the liver. In response to decreased arterial pressure or plasma sodium levels, the kidney releases renin, which cleaves AGT to generate angiotensin I (ATI). ATI then undergoes conversion through two pathways: via angiotensin-converting enzyme (ACE; also known as ACE1) to form angiotensin II (ATII), or via angiotensin-converting enzyme 2 (ACE2) to produce angiotensin 1–7 (Ang1–7). ATII can undergo further sequential conversions to form angiotensin III (ATIII) and angiotensin IV (ATIV) by aminopeptidases A and N, respectively. Alternative enzymatic pathways involving cathepsins B, D, and G, chymostatin-sensitive ATII-generating enzyme, and chymase can also generate these angiotensin peptides. The binding of these peptides to their respective receptors triggers cellular signaling cascades with ATII binding to ATII receptor 1 (AT_1_R) and ATII receptor 2 (AT_2_R), being significant in blood pressure regulation through smooth muscle contraction and aldosterone release [31].

Research continues to reveal the complexity of the renin–angiotensin system and its role in physiological and pathological processes, particularly in tumor biology through its paracrine function [44]. The paracrine renin–angiotensin system, also known as the local renin–angiotensin system, functions locally within specific tissues and organs, in contrast to the classical endocrine renin–angiotensin system that acts systemically [29]. Components of the renin–angiotensin system are expressed locally in many tumors by cancer cells, immune cells, and stromal cells [45]. The paracrine renin–angiotensin system is implicated in cellular proliferation, inflammation, invasion and adhesion, angiogenesis, endothelial-to-mesenchymal transition (EMT), and cancer metastasis [46]. Further, signal transduction of ATII in the paracrine renin–angiotensin system involves a wide array of signaling pathways and pathophysiological functions. These include inflammation; end-organ damage associated with aging; metabolic dysfunction; cardiovascular, neurological, and kidney disease; and cancer [47]. AT_1_R activation contributes to inflammation by producing reactive oxygen species (ROS) via NADPH oxidase activation, enabling ATII to function as an inflammatory cytokine [48]. Additionally, AT_1_R activation induces pro-fibrotic effects and hypertrophy [47]. Conversely, AT_2_R activation antagonizes the effects of AT_1_R, causing vasodilation and exerting anti-proliferative, anti-fibrotic, and anti-inflammatory effects, while also reducing sodium excretion [47]. PRR, a key component of the renin–angiotensin system, also promotes fibrosis and renal dysfunction [47]. Renin–angiotensin system dysregulation is common in many cancer types, including melanoma, with experimental models demonstrating that renin–angiotensin system inhibition reduces tumor growth, angiogenesis, and metastasis [49].

### 3.2. Renin–Angiotensin System Regulation of Cancer Stem Cells in Melanoma

The paracrine renin–angiotensin system plays an important role in regulating cellular differentiation across multiple cell types [50,51], including stem cell maintenance and progenitor cell differentiation in hematopoiesis [52], vasculogenesis [53], myeloid differentiation [54], and erythropoiesis [55]. This is relevant, as metastatic melanoma express components of the renin–angiotensin system: PRR, AT_2_R, and ACE, with ACE localizing to the endothelium of tumor microvessels [34,35].

Further, SOX2^+^/OCT4^+^ phenotypic CSCs in metastatic melanoma to the brain [6] and the head and neck lymph nodes [34] express components of the renin–angiotensin system, a pathway well described in brain tissue [56]. Additional research is needed to determine the precise regulatory impact of paracrine renin–angiotensin system signaling on these CSCs, which are proposed drivers of melanoma and treatment resistance [57].

The renin–angiotensin system and the Wnt/β pathway demonstrate bidirectional interaction. While PRR may induce Wnt/β-catenin [58], Wnt pathway activation affects components of the renin–angiotensin system [59]. Given the established role of Wnt signaling in the regulation of ESCs and the pathogenesis of melanoma, further investigation is warranted to elucidate how renin–angiotensin system modulation affects Wnt pathway activity and CSC properties in the melanoma TME. One study investigating colon adenocarcinoma-derived primary cell lines with CSC-like phenotypes shows that renin–angiotensin system inhibition reduces pluripotency marker expression and tumorsphere forming capacity [60]. Mutations in genes involved in the canonical Wnt-signaling pathway occur in up to 10% of melanoma cases, contributing to its dysregulation [61]. Wnt/β-catenin signaling alters renin–angiotensin system gene expression in various disease contexts, with β-catenin overexpression in tubular epithelial cells inducing mRNA expression of AGT, ACE, renin, AT_1_R, and AT_2_R [59]. ACE inhibition has been shown to downregulate Wnt target genes in colorectal cancer [62], suggesting that targeting the upstream Wnt/β-catenin pathway alongside the paracrine renin–angiotensin system may be necessary. In melanoma stem-like cells, NOP14 nucleolar protein suppresses cell stemness and function through Wnt/β-catenin signaling inactivation [63]. These findings suggest a potential role for the Wnt/β-catenin pathway in both the paracrine renin-angiotensin system within the melanoma TME, and in regulating melanoma CSC stemness.

Recently, a tumor suppressor function for *AGTR1* has been suggested. This is supported by heightened CpG island methylation of *AGTR1* and its reduced expression in metastatic melanoma compared to primary melanoma [64]. AT_1_R inhibition promotes melanoma cell proliferation in serum-free conditions [64].

The paracrine renin–angiotensin system influences the TME through its effect on immune function [65], interacting with three key classes of immunosuppressive cells [65]. Cancer-associated fibroblasts express components of the renin–angiotensin system, including AT_1_R [45], which upon activation leads to TGF-β formation, stimulating EMT and promoting metastasis and treatment resistance [66,67]. Myeloid-derived suppressor cells inhibit CD8^+^ T cell activation [65], with ACE being implicated in their accumulation within the TME [68]. Tumor-associated macrophages of the M2 phenotype are tumorigenic, with ACE regulating their production of inflammatory cytokines [65]; notably, ACE inhibition with the ACE inhibitor captopril increases Kupffer cell infiltration in liver metastasis from colorectal cancer [69]. The presence of these immunosuppressive cells within the melanoma TME [70], and their interaction with the paracrine renin–angiotensin system, highlight possible therapeutic avenues. Further exploration of the effects of renin–angiotensin system inhibition on melanoma TME may yield valuable insights into novel treatment strategies.

**Figure 3:**
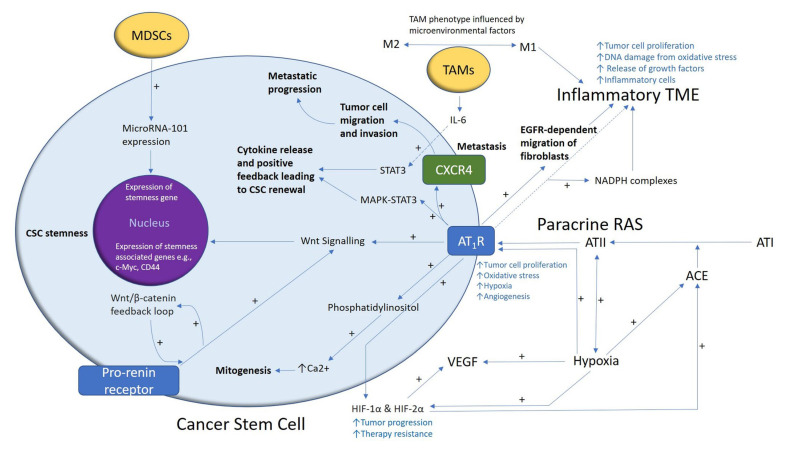
A proposed model demonstrating the role of the paracrine renin–angiotensin system (RAS) in the cancer stem cell (CSC) niche. A CSC (with the cytoplasm in light blue and the nucleus in purple) residing within the tumor microenvironment (TME). Angiotensin II (ATII) activates ATII receptor 1 (AT_1_R), promoting the hallmarks of cancer, including creating an inflammatory state through multiple mechanisms. AT_1_R activates phosphatidylinositol signaling, which increases cytosolic Ca^2+^ to promote mitogenesis. Hypoxia increases paracrine RAS activity by upregulating angiotensin-converting enzyme (ACE) and the expression of hypoxia-inducible factor 1α (HIF-1α) and HIF-2α, which increase tumor progression and treatment resistance. HIF-1α, HIF-2α, and hypoxia increase the expression of vascular endothelial growth factor (VEGF) which increases angiogenesis. The binding of AT_1_R to C-X-C chemokine receptor type 4 (CXCR4) promotes tumor cell migration and invasion, leading to metastatic progression. AT_1_R, via MAPK-STAT3 signaling, contributes to a cytokine release that leads to CSC renewal. AT_1_R signaling also contributes to the migration of fibroblasts in an epidermal growth factor receptor (EGFR)-dependent fashion. AT_1_R signaling and the pro-renin receptor, which act in a feedback loop with Wnt/β-catenin, increase Wnt signaling, which promotes CSC stemness by upregulating stemness-associated markers. Myeloid-derived suppressor cells (MDSCs) promote CSC characteristics by increasing microRNA-101 expression that induces expression of stemness-related genes in CSCs. Under the influence of the TME, the polarization of tumor-associated macrophages (TAMs)—immune cells that are located within the TME—changes from the M1 to the M2 phenotype. M2 TAMs induce the proliferation of CSCs via interleukin 6 (IL-6)-induced activation of STAT3, leading to cytokine release and positive feedback contributing to CSC renewal. Abbreviations: ATI, angiotensin I; MAPK, mitogen-activated protein kinase. Reproduced from *Journal of Histochemistry and Cytochemistry* [31].

We propose, within the TME, that CSCs are regulated by paracrine renin–angiotensin signaling (Figure 3) [31]. ATII, the physiologically active end-product of this system, activates AT_1_R to promote the hallmarks of cancer: tumor cell proliferation, oxidative stress, hypoxia, angiogenesis, and inflammation [71]. This contributes to an inflammatory TME by increasing the number of NADPH complexes, leading to tumor cell proliferation, DNA damage from oxidative stress, and growth factor release [31,72]. AT_1_R also stimulates mitogenesis through phosphatidylinositol-mediated calcium signaling.

Hypoxia increases paracrine renin–angiotensin system signaling by upregulating ACE and the expression of hypoxia-inducible factor 1α (HIF-1α) and HIF-2α, leading to treatment resistance and increased angiogenesis through elevated vascular endothelial growth factor expression [73]. AT_1_R interaction with C-X-Chemokine receptor type 4 promotes metastasis [74]. AT_1_R signaling and the PRR, which act in a feedback loop with Wnt/β-catenin, increase Wnt signaling which may promote CSC stemness by upregulating stemness-associated markers [31]. Within the TME, myeloid-derived suppressor cells enhance CSC characteristics through increased microRNA-101 expression, which increases the expression of the stemness genes of CSCs [75]. M2 tumor-associated macrophages stimulate CSC proliferation through interleukin-6/STAT3 signaling [31,76]. Together, these signaling pathways within the TME demonstrate the complex interplay between the renin–angiotensin system and the various cellular components that contribute to cancer progression and the possible maintenance of the CSC phenotype. Further research is needed to determine whether these observations translate to the melanoma TME.

### 3.3. The Renin–Angiotensin System and Melanoma Metastasis

There is increasing evidence of the role of the renin–angiotensin system in cancer metastasis. Martínez-Meza et al. [77] demonstrate that AT_2_R activation inhibits transendothelial migration, cellular migration, and metastasis in vivo. These effects are completely abrogated upon AT_2_R silencing using shRNA. Notably, AT_2_R activation reduces the lung metastasis of B16F10 (cav-1) melanoma cells in C57BL/6 mice, which is attributed to increased non-receptor tyrosine phosphatase 1B activity [77].

Ishikane et al. [78] observe that ATII treatment increases lung metastasis in C57BL/6 mice injected with B16/F10 murine melanoma cells compared to vehicle-treated controls. This effect is inhibited by the angiotensin receptor blocker (ARB) valsartan. Conversely, in *AGTR1α* knockout mice, ATII-mediated melanoma lung metastases are significantly reduced. Furthermore, ATII upregulates mRNA expression of E-selectin, promoting melanoma cell adherence to the vascular endothelium [78]. Despite these findings, investigation into the use of renin–angiotensin system inhibitors in the treatment of metastatic melanoma in humans remains limited.

Renin–angiotensin system inhibition may affect not only AT_1_R and AT_2_R signaling, but also its interactions with the overactive Ras/RAF/MAPK/ERK and PI3K/AKT/mTOR pathways in melanoma. The observation that AT_2_R antagonists potentiate the effects of BRAF and MEK inhibitors underscores the potential benefits of combining renin–angiotensin system inhibitors with targeted therapies for melanoma. Further research is needed to elucidate the precise mechanisms by which renin–angiotensin system inhibitors block these signaling pathways to enhance the durability of current targeted therapy.

### 3.4. The Renin–Angiotensin System Interacts with the Ras/RAF/MAPK/ERK and PI3K/AKT/mTOR Pathways

**Figure 4:**
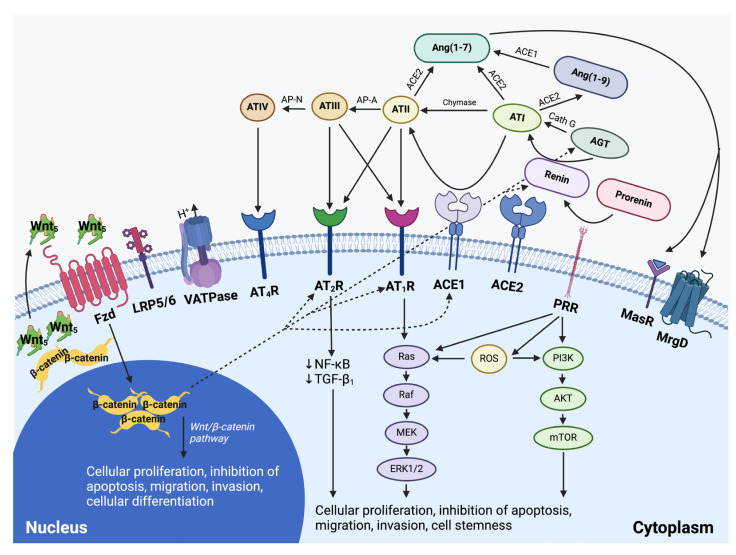
A schema showing the effect of the paracrine renin–angiotensin system and its convergent signaling pathways on the tumor microenvironment, and its influence on cellular proliferation, invasiveness, and cell survival in cancer development. The renin–angiotensin system interacts with downstream pathways, such as the Ras/RAF/MEK/ERK pathway (light purple) and the PI3K/AKT/mTOR pathway (light green), and the upstream Wnt/β-catenin pathway that influences cellular proliferation, migration, inhibition of apoptosis, migration, and invasion. It also influences gene expression of components of the renin–angiotensin system (see text). PRR, pro-renin receptor; LRP6, low-density lipoprotein receptor-related protein; Fzd, frizzled receptor; Cath G, cathepsin G; Cath B, cathepsin B; Cath D, cathepsin D; ACE1, angiotensin-converting enzyme 1; ACE2, angiotensin-converting enzyme 2; ADP, adenosine diphosphate; AGT, angiotensinogen; ATP, adenosine triphosphate; Ang(1–7), angiotensin (1–7); Ang(1–9), angiotensin (1–9); AP-A, aminopeptidase-A; NEP, neutral endopeptidase; AP-N, aminopeptidase-N; ATI, angiotensin I; ATII, angiotensin II; ATIII, angiotensin III; ATIV, angiotensin IV; AT_1_R, angiotensin II receptor 1; AT_2_R, angiotensin II receptor 2; AT_4_R, angiotensin II receptor 4; MrgD, Mas-related-G protein coupled receptor; MasR, Mas receptor; mTOR, mammalian target of rapamycin; NF-κB, nuclear factor kappa B; TGF-β1, transforming growth factor-β1; V-ATPase, vacuolar H+-adenosine triphosphate. Reproduced and adapted with permission from *Biomedicines* [13].

The renin–angiotensin system interacts with the Ras/RAF/MAPK/ERK and PI3K/AKT/mTOR signaling pathways [79] (Figure 4). PRR-induced upregulation of ERK1/2 leads to the increased production of transforming growth factor-β, cellular proliferation, mediating cancer progression, and metastasis [80,81]. Through an ATII-independent mechanism, PRR also induces ROS formation, further enhancing PI3K/AKT/mTOR and Ras/RAF/MAPK/ERK signaling [80,82]. Furthermore, renin–angiotensin system inhibitors potentiate the effect of BRAF and MEK inhibitors in *BRAFV600*-mutated melanoma cells. AT_2_R antagonists enhance the efficacy of these inhibitors, while reducing angiogenesis and melanoma growth [64]. Conversely, AT_2_R agonists induce proliferation under serum-free conditions [64]. Together, these findings suggest a potential role for renin–angiotensin system regulation in the treatment of melanoma. Further research is required to determine if this interaction impacts CSCs in melanoma.

Melanoma demonstrates significant heterogeneity and resistance to conventional therapies, which has been attributed to the presence of CSCs that drive tumorigenesis and cancer progression and recurrence. The paracrine renin–angiotensin system within the melanoma TME may play an important role in maintaining melanoma CSCs through its interaction with the Ras/RAF/MAPK/ERK and PI3K/AKT/mTOR pathways. The dysregulated paracrine renin–angiotensin system, especially through the binding of ATII to AT_1_R, may enhance melanoma CSC properties by activating the Ras/RAF/MAPK/ERK and PI3K/AKT/mTOR pathways, ultimately leading to the increased expression of CSC markers and self-renewal capacity. The convergence of the renin–angiotensin system onto the Ras/RAF/MAPK/ERK and PI3K/AKT/mTOR pathways in melanoma presents exciting research avenues for therapeutic interventions. Targeting the paracrine renin–angiotensin system may disrupt signaling critical for CSC maintenance, thereby halting melanoma progression, treatment resistance, and recurrence. Targeting this broader network of interacting pathways, rather than individual pathways singly, may prove more effective in regulating melanoma CSCs, leading to improved outcomes for patients with advanced melanoma.

## 4. Improving the Effectiveness of Immunotherapy by Targeting the Renin–Angiotensin System and Cancer Stem Cells in Melanoma

Recent advances in immunotherapy have significantly improved the survival outcomes of patients with advanced melanoma, as evidenced by the approximately 50% 5-year survival rate of patients with stage IV melanoma treated with combination immunotherapy [83]. The effectiveness of immunotherapy in melanoma is partly attributed to its ability to counteract tumor-induced immunosuppression, a hallmark of metastatic melanoma. This immunosuppression results from persistent antigenic stimulation of the immune system and elevated expression of inhibitory receptors on tumor antigen-specific T cells [83].

The first immunotherapy agent, ipilimumab, approved by the FDA in 2011 for unresectable stage IV melanoma, shows a median overall survival of 10.1 months, with associated grade ≥3 adverse effects in 10–15% of patients [21]. In 2015, the anti-PD1 antibody pembrolizumab became a new standard of care for patients with ipilimumab-resistant melanoma [84]. Patients treated with a combination of nivolumab and ipilimumab for stages III or IV melanoma have a 52% 5-year overall survival [85]. A recent phase II clinical trial shows pembrolizumab, as a neoadjuvant treatment in patients with resected stages III or IV melanoma, significantly prolongs event-free survival at 2 years (72%, 95% CI, 64 to 80), compared to those receiving adjuvant pembrolizumab alone (49%, 95% CI, 41 to 59) [79]. This underscores the benefit of neoadjuvant immunotherapy for this group of patients [86].

Despite advances in immunotherapy, treatment resistance, recurrence, and adverse effects remain significant challenges. Recent research has targeted alternative immune checkpoints. The RELATIVITY-047 phase II/III clinical trial has demonstrated that simultaneous targeting lymphocyte-activation gene 3 and PD-1 with relatimab and nivolumab, respectively, provides greater survival benefits compared to targeting PD-1 alone [87]. Notwithstanding the outcomes seen with immunotherapy, significant adverse effects are observed in both mono- and combination immunotherapy. In the RELATIVITY-047 trial, grades three or four treatment-related adverse effects were observed in 18.9% of patients in the combination treatment group, compared to 9.7% in those receiving nivolumab alone [87].

Emerging research suggests that targeting the paracrine renin–angiotensin system improves the efficacy of anti-PD1 antibodies [88]. This effect is achieved via decreased renin–angiotensin system-stimulated fibroblast CC motif chemokine ligand 5 production in melanoma-transplanted mice. Recent studies have also highlighted the role of cancer stemness in immunotherapy resistance. Using a large publicly available dataset, a signature Stem.Sig has been developed to predict which patients will benefit from immunotherapy, based on the association between cancer stemness and resistance to checkpoint inhibitors [89]. This study demonstrates that cells with cancer stemness are less likely to respond to checkpoint inhibitors. This supports the notion that immunotherapy resistance in melanoma may be attributable to CSCs. Future research into immunotherapy for melanoma may involve specific targeting of CSCs alongside immunotherapy to improve survival outcomes.

## 5. Cancer Stem Cells and Dysregulated Pathways in Melanoma

In health, the Ras/RAF/MAPK/ERK and PI3K/AKT/mTOR signaling pathways are integral to multiple processes regulating cellular function, including cell growth, survival, and nutrient intake [40]. These pathways are dysregulated in melanoma, contributing to the maintenance and self-renewal of CSCs. The expression of PRR in melanoma may contribute to this dysregulation through the production of ROS, although further research is needed to fully elucidate this.

Targeting these pathways, particularly mTOR signaling to overcome the inevitable acquired resistance to BRAF and MEK inhibition in melanoma, has been investigated. The mTOR inhibitors rapamycin (sirolimus) and NVP-BEZ235 induce apoptosis and cell cycle arrest in *BRAF* mutant melanoma cell lines [90]. Notably, metformin, a widely used anti-diabetic medication that targets APK/mTOR signaling, reduces cellular proliferation and displays anti-tumor properties across multiple cancer types [91]. Studies in melanoma have shown that metformin inhibits melanoma development in vivo, with three of the seven metformin-treated mice showing no measurable tumors [91].

**Figure 5:**
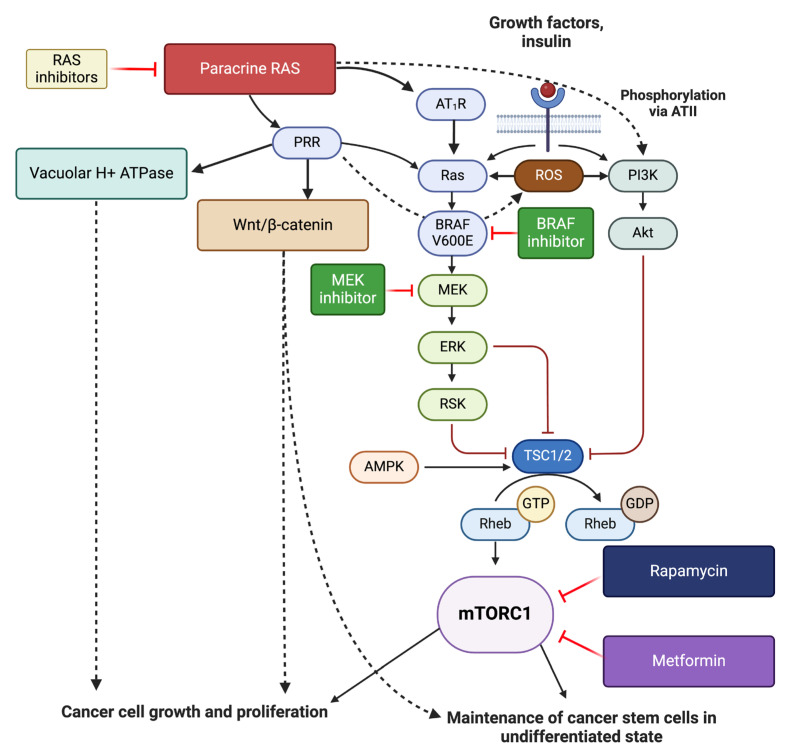
A schema showing how the Ras/RAF/MAPK/ERK and PI3K/AKT/mTOR pathways can be inhibited in melanoma. BRAF can be targeted by BRAF inhibitors, and MEK by MEK inhibitors. Pro-renin receptor (PRR) and angiotensin II receptor 1 (AT_2_R), which activate Ras, can be inhibited through inhibition of the paracrine renin–angiotensin system (RAS). PRR also increases the production of reactive oxygen species (ROS), which contributes to the overactivation of the Ras/RAF/MAPK/ERK and PI3K/AKT/mTOR pathways. PRR also acts on vacuolar ATPase and Wnt/β-catenin signaling to facilitate cancer progression. Angiotensin II (ATII) induces phosphorylation of PI3K/AKT, driving tumor progression. mTORC1 signaling can be inhibited by rapamycin (sirolimus) and metformin. Figure created using Biorender.

PRR may contribute to CSC maintenance through its interaction with multiple signaling pathways, including Ras/RAF/MAPK/ERK, PI3K/AKT/mTOR, vacuolar H+ ATPase, and Wnt/β-catenin [80], promoting aberrant cellular proliferation and maintaining cells in the undifferentiated state (Figure 5). Additionally, ATII induces PI3K/AKT phosphorylation, leading to increased NF-κB activity, which promotes cancer progression, metastasis, and resistance to chemotherapy and radiotherapy [80,92]. The complex interplay between these pathways in melanoma presents interesting research avenues for therapeutic intervention.

**Figure 6:**
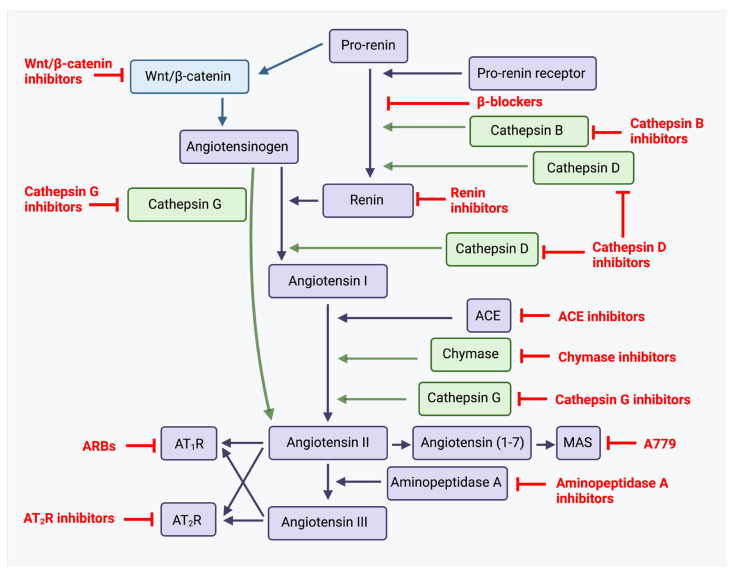
The renin–angiotensin system and its bypass loops and converging signaling pathways can be targeted at different points. The renin–angiotensin system (black) regulates blood pressure, stem cell differentiation, and tumor development. Bypass loops in the system involving cathepsins B, D, and G, and chymase (green) provide redundancy. Multiple points of the pathway can be targeted by specific inhibitors (red). ACE, angiotensin-converting enzyme; ARBs, AT_1_R blockers. Adapted with permission from *Frontiers in Oncology* [93]. Diagram recreated with BioRender.com, accessed on 13 June 2024.

Current treatment of advanced melanoma typically consists of single agents. Given the presence of numerous inter-related treatment targets (Figure 5), simultaneous targeting of the Ras/RAF/MAPK/ERK pathway and/or the PI3K/AKT/mTOR pathway with targeted therapy such as a MEK or BRAF inhibitor in melanoma, and inhibition of the renin–angiotensin system, or direct inhibition of PRR, may result in more effective suppression of these overactive pathways. Investigation into the possible benefits of inhibiting the paracrine renin–angiotensin system and its related pathways at multiple points using pre-existing, low-cost, well-tolerated commonly available oral medications presents an exciting prospect (Figure 6) [13,93]. Table 1 highlights the results of some of the pre-clinical and clinical studies on inhibition of mTOR, MEK, BRAF, and the renin–angiotensin system in melanoma.

## 6. Conclusions

While targeted therapy and immunotherapy have improved survival outcomes in patients with advanced melanoma, challenges such as treatment resistance, adverse effects, and prohibitive cost persist. This review highlights the complex interplay between CSCs, the TME, the paracrine renin–angiotensin system, and dysregulated signaling pathways, in melanoma. The expression of components of the renin–angiotensin system by phenotypic CSCs in metastatic melanoma, coupled with the interactions between the renin–angiotensin system and the overactive Ras/RAF/MAPK/ERK and PI3K/AKT/mTOR pathways, present vast therapeutic possibilities. The potential of renin–angiotensin system inhibition with low-cost, well-tolerated, and commonly available oral medications to enhance the efficacy of current therapies, including BRAF and MEK inhibitors and immunotherapy agents, warrants further investigation. The role of cancer stemness in immunotherapy resistance underscores the need to develop therapeutic strategies to target CSCs in melanoma. Combined treatment involving renin–angiotensin system inhibitors, targeted therapy, and/or immunotherapy, and possibly mRNA vaccines may offer more a durable treatment for advanced melanoma. Future research should focus on elucidating the mechanisms by which the paracrine renin–angiotensin system influences the TME and CSCs.

## Figures and Tables

**Table 1:** Targeting mTOR, MEK, BRAF, and the renin–angiotensin system in melanoma.

Target	Findings	Study type
mTOR signaling	mTOR inhibitor rapamycin, with the RAF inhibitor sorafenib suppress invasive melanoma growth in vitro [94].	Pre-clinical
	The PI3K-mTOR dual inhibitor VS-5584 inhibits survival and proliferation of primary human melanoma cells in vitro and in vivo [95].	Pre-clinical
Renin–angiotensin system	Inhibiting angiotensin II receptor 1 decreases melanoma tumor growth and tumor-associated microvessel density in vivo [49].	Pre-clinical
	Inhibiting the renin–angiotensin system improves the efficacy of anti-PD1 antibodies in vivo [88].	Pre-clinical
MEK and BRAF	Trametinib (a MEK inhibitor) improves rates of progression-free and overall survival in patients with metastatic melanoma [15].	Clinical trial
	Treatment of patients with metastatic melanoma carrying the V600E BRAF mutation with Vemurafenib causes complete or partial regression of tumor [96].	Clinical trial
	A combination of dabrafenib (a BRAF inhibitor) and trametinib improves the rate of progression-free survival in patients with metastatic melanoma [97].	Clinical trial

## Abbreviations

The following abbreviations are used in this manuscript:

AGT	Angiotensinogen
ATI	Angiotensin I
ATII	Angiotensin II
ACE	Angiotensin-converting enzyme
ACE2	Angiotensin-converting enzyme 2
ARB	Angiotensin receptor blocker
ATIII	Angiotensin III
ATIV	Angiotensin IV
AT1R	Angiotensin receptor 1
AT2R	Angiotensin receptor 2
CAFs	Cancer-associated fibroblasts
CSC	Cancer stem cell
CTLA-4	Cytotoxic T-lymphocyte-associated protein-4
EMT	Endothelial-to-mesenchymal transition
ESCs	Embryonic stem cells
LAG-3	Lymphocyte-activation gene-3
MDSCs	Myeloid-derived suppressor cells
PD-1	Programmed cell death protein-1
PRR	Pro-renin receptor
ROS	Reactive oxygen species
TAMs	Tumor-associated macrophages
TME	Tumor microenvironment

## References

[1] Garbe C., Amaral T., Peris K., Hauschild A., Arenberger P., Basset-Seguin N., Bastholt L., Bataille V., del Marmol V., Dréno B. (2022). European consensus-based interdisciplinary guideline for melanoma. Part 1: Diagnostics: Update 2022. Eur. J. Cancer.

[2] Whiteman D.C., Green A.C., Olsen C.M. (2016). The growing burden of invasive melanoma: Projections of incidence rates and numbers of new cases in six susceptible Populations through 2031. J. Investig. Dermatol..

[3] Patel J., Didolkar M., Pickren J., Moore R. (1978). Metastatic pattern of malignant melanoma. Am. J. Surg..

[4] Yoganandarajah V., Patel J., van Schaijik B., Bockett N., Brasch H.D., Paterson E., Sim D., Davis P.F., Roth I.M., Itinteang T. (2020). Identification of Cancer Stem Cell Subpopulations in Head and Neck Metastatic Malignant Melanoma. Cells.

[5] Sangster A.B., Chang-McDonald B., Patel J., Bockett N., Paterson E., Davis P.F., Tan S.T. (2021). Expression of cathepsins B and D by cancer stem cells in head and neck metastatic malignant melanoma. Melanoma Res..

[6] Wickremesekera A.C., Brasch H.D., Lee V.M., Davis P.F., Woon K., Johnson R., Tan S.T., Itinteang T. (2018). Expression of cancer stem cell markers in metastatic melanoma to the brain. J. Clin. Neurosci..

[7] Marzagalli M., Raimondi M., Fontana F., Marelli M.M., Moretti R.M., Limonta P. (2019). Cellular and molecular biology of cancer stem cells in melanoma: Possible therapeutic implications. Semin. Cancer Biol..

[8] Batlle E., Clevers H. (2017). Cancer stem cells revisited. Nat. Med..

[9] Loh J.-J., Ma S. (2024). Hallmarks of cancer stemness. Cell Stem Cell.

[10] Eyler C.E., Rich J.N. (2008). Survival of the Fittest: Cancer Stem Cells in Therapeutic Resistance and Angiogenesis. J. Clin. Oncol..

[11] Gupta G., Merhej G., Saravanan S., Chen H. (2022). Cancer resistance to immunotherapy: What is the role of cancer stem cells?. Cancer Drug Resist..

[12] Shackleton M., Quintana E., Fearon E.R., Morrison S.J. (2009). Heterogeneity in Cancer: Cancer Stem Cells versus Clonal Evolution. Cell.

[13] Kilmister E.J., Koh S.P., Weth F.R., Gray C., Tan S.T. (2022). Cancer Metastasis and Treatment Resistance: Mechanistic Insights and Therapeutic Targeting of Cancer Stem Cells and the Tumor Microenvironment. Biomedicines.

[14] Strashilov S., Yordanov A. (2021). Aetiology and Pathogenesis of Cutaneous Melanoma: Current Concepts and Advances. Int. J. Mol. Sci..

[15] Flaherty K.T., Robert C., Hersey P., Nathan P., Garbe C., Milhem M., Demidov L.V., Hassel J.C., Rutkowski P., Mohr P. (2012). Improved Survival with MEK Inhibition in BRAF-Mutated Melanoma. N. Engl. J. Med..

[16] Hauschild A., Grob J.-J., Demidov L.V., Jouary T., Gutzmer R., Millward M., Rutkowski P., Blank C.U., Miller W.H., Kaempgen E. (2012). Dabrafenib in BRAF-mutated metastatic melanoma: A multicentre, open-label, phase 3 randomised controlled trial. Lancet.

[17] Guo W., Wang H., Li C. (2021). Signal pathways of melanoma and targeted therapy. Signal Transduct. Target. Ther..

[18] Huang A.C., Zappasodi R. (2022). A decade of checkpoint blockade immunotherapy in melanoma: Understanding the molecular basis for immune sensitivity and resistance. Nat. Immunol..

[19] Maurer D.M., Butterfield L.H., Vujanovic L. (2019). Melanoma vaccines: Clinical status and immune endpoints. Melanoma Res..

[20] Khattak A., Weber J.S., Meniawy T., Taylor M.H., Ansstas G., Kim K.B., McKean M., Long G.V., Sullivan R.J., Faries M.B. (2023). Distant metastasis-free survival results from the randomized, phase 2 mRNA-4157-P201/KEYNOTE-942 trial. J. Clin. Oncol..

[21] Hodi F.S., O’Day S.J., McDermott D.F., Weber R.W., Sosman J.A., Haanen J.B., Gonzalez R., Robert C., Schadendorf D., Hassel J.C. (2010). Improved Survival with Ipilimumab in Patients with Metastatic Melanoma. N. Engl. J. Med..

[22] Robert C., Ribas A., Schachter J., Arance A., Grob J.-J., Mortier L., Daud A., Carlino M.S., McNeil C.M., Lotem M. (2019). Pembrolizumab versus ipilimumab in advanced melanoma (KEYNOTE-006): Post-hoc 5-year results from an open-label, multicentre, randomised, controlled, phase 3 study. Lancet Oncol..

[23] Robert C., Schachter J., Long G.V., Arance A., Grob J.J., Mortier L., Daud A., Carlino M.S., McNeil C., Lotem M. (2015). Pembrolizumab versus Ipilimumab in Advanced Melanoma. N. Engl. J. Med..

[24] Weber J., Mandalà M., Del Vecchio M., Gogas H.J., Arance A.M., Cowey C.L., Dalle S., Schenker M., Chiarion-Sileni V., Marquez-Rodas I. (2017). Adjuvant Nivolumab versus Ipilimumab in Resected Stage III or IV Melanoma. N. Engl. J. Med..

[25] Eggermont A.M.M., Blank C.U., Mandalà M., Long G.V., Atkinson V.G., Dalle S., Haydon A.M., Meshcheryakov A., Khattak A., Carlino M.S. (2021). Adjuvant pembrolizumab versus placebo in resected stage III melanoma (EORTC 1325-MG/KEYNOTE-054): Distant metastasis-free survival results from a double-blind, randomised, controlled, phase 3 trial. Lancet Oncol..

[26] Eggermont A.M.M., Blank C.U., Mandala M., Long G.V., Atkinson V.G., Dalle S., Haydon A.M., Meshcheryakov A., Khattak A., Carlino M.S. (2020). Longer Follow-Up Confirms Recurrence-Free Survival Benefit of Adjuvant Pembrolizumab in High-Risk Stage III Melanoma: Updated Results from the EORTC 1325-MG/KEYNOTE-054 Trial. J. Clin. Oncol..

[27] Luke J.J., Rutkowski P., Queirolo P., Del Vecchio M., Mackiewicz J., Chiarion-Sileni V., Merino L.d.l.C., A Khattak M., Schadendorf D., Long G.V. (2022). Pembrolizumab versus placebo as adjuvant therapy in completely resected stage IIB or IIC melanoma (KEYNOTE-716): A randomised, double-blind, phase 3 trial. Lancet.

[28] Ascierto P.A., Dummer R., Gaudy-Marqueste C., Bowyer S., Lipson E.J., Ghisoni E., Middleton M.R., Ratto B., Jackson W.J., Cheong A. (2024). Efficacy and safety of triplet nivolumab, relatlimab, and ipilimumab (NIVO + RELA + IPI) in advanced melanoma: Results from RELATIVITY-048. J. Clin. Oncol..

[29] Paul M., Mehr A.P., Kreutz R. (2006). Physiology of Local Renin-Angiotensin Systems. Physiol. Rev..

[30] Catarata M.J., Ribeiro R., Oliveira M.J., Cordeiro C.R., Medeiros R. (2020). Renin-Angiotensin System in Lung Tumor and Microenvironment Interactions. Cancers.

[31] Kilmister E.J., Tan S.T. (2021). The Role of the Renin–Angiotensin System in the Cancer Stem Cell Niche. J. Histochem. Cytochem..

[32] Zhou S., Lu J., Liu S., Shao J., Liu Z., Li J., Xiao W. (2023). Role of the tumor microenvironment in malignant melanoma organoids during the development and metastasis of tumors. Front. Cell Dev. Biol..

[33] Kapoor-Narula U., Lenka N. (2022). Cancer stem cells and tumor heterogeneity: Deciphering the role in tumor progression and metastasis. Cytokine.

[34] Siljee S., Pilkington T., Brasch H.D., Bockett N., Patel J., Paterson E., Davis P.F., Tan S.T. (2020). Cancer Stem Cells in Head and Neck Metastatic Malignant Melanoma Express Components of the Renin-Angiotensin System. Life.

[35] Wickremesekera A.C., Brasch H.D., Lee V.M., Davis P.F., Parker A., Koeck H., Itinteang T., Tan S.T. (2019). Cancer stem cell subpopulations in metastatic melanoma to the brain express components of the renin-angiotensin system. J. Cancer Metastasis Treat..

[36] Caporali S., Alvino E., Lacal P.M., Levati L., Giurato G., Memoli D., Caprini E., Cappellini G.C.A., D’atri S. (2016). Targeting the PI3K/AKT/mTOR pathway overcomes the stimulating effect of dabrafenib on the invasive behavior of melanoma cells with acquired resistance to the BRAF inhibitor. Int. J. Oncol..

[37] Murakami M., Ichisaka T., Maeda M., Oshiro N., Hara K., Edenhofer F., Kiyama H., Yonezawa K., Yamanaka S. (2004). mTOR Is Essential for Growth and Proliferation in Early Mouse Embryos and Embryonic Stem Cells. Mol. Cell. Biol..

[38] Bi L., Okabe I., Bernard D.J., Wynshaw-Boris A., Nussbaum R.L. (1999). Proliferative Defect and Embryonic Lethality in Mice Homozygous for a Deletion in the p110α Subunit of Phosphoinositide 3-Kinase. J. Biol. Chem..

[39] Peng X.-D., Xu P.-Z., Chen M.-L., Hahn-Windgassen A., Skeen J., Jacobs J., Sundararajan D., Chen W.S., Crawford S.E., Coleman K.G. (2003). Dwarfism, impaired skin development, skeletal muscle atrophy, delayed bone development, and impeded adipogenesis in mice lacking Akt1 and Akt2. Genes. Dev..

[40] Yu J.S.L., Cui W. (2016). Proliferation, survival and metabolism: The role of PI3K/AKT/mTOR signalling in pluripotency and cell fate determination. Development.

[41] Craig S., Earnshaw C.H., Virós A. (2018). Ultraviolet light and melanoma. J. Pathol..

[42] Czarnecka A.M., Bartnik E., Fiedorowicz M., Rutkowski P. (2020). Targeted Therapy in Melanoma and Mechanisms of Resistance. Int. J. Mol. Sci..

[43] Kozar I., Margue C., Rothengatter S., Haan C., Kreis S. (2019). Many ways to resistance: How melanoma cells evade targeted therapies. Biochim. Biophys. Acta. Rev. Cancer.

[44] Afsar B., Afsar R.E., Ertuglu L.A., Kuwabara M., Ortiz A., Covic A., Kanbay M. (2020). Renin-angiotensin system and cancer: Epidemiology, cell signaling, genetics and epigenetics. Clin. Transl. Oncol..

[45] Nakamura K., Yaguchi T., Ohmura G., Kobayashi A., Kawamura N., Iwata T., Kiniwa Y., Okuyama R., Kawakami Y. (2017). Involvement of local renin-angiotensin system in immunosuppression of tumor microenvironment. Cancer Sci..

[46] Almutlaq M., Alamro A.A., Alamri H.S., Alghamdi A.A., Barhoumi T. (2021). The Effect of Local Renin Angiotensin System in the Common Types of Cancer. Front. Endocrinol..

[47] Forrester S.J., Booz G.W., Sigmund C.D., Coffman T.M., Kawai T., Rizzo V., Scalia R., Eguchi S. (2018). Angiotensin II Signal Transduction: An Update on Mechanisms of Physiology and Pathophysiology. Physiol. Rev..

[48] Dikalova A., Clempus R., Lasseègue B., Cheng G., McCoy J., Dikalov S., San Martin A., Lyle A., Weber D.S., Weiss D. (2005). Nox1 Overexpression Potentiates Angiotensin II-Induced Hypertension and Vascular Smooth Muscle Hypertrophy in Transgenic Mice. Circulation.

[49] Otake A.H., Mattar A.L., Freitas H.C., Machado C.M.L., Nonogaki S., Fujihara C.K., Zatz R., Chammas R. (2009). Inhibition of angiotensin II receptor 1 limits tumor-associated angiogenesis and attenuates growth of murine melanoma. Cancer Chemother. Pharmacol..

[50] Sadik N.A.-H., Metwally N.S., Shaker O.G., Soliman M.S., Mohamed A.A., Abdelmoaty M.M. (2017). Local renin-angiotensin system regulates the differentiation of mesenchymal stem cells into insulin-producing cells through angiotensin type 2 receptor. Biochimie.

[51] Matsushita K., Wu Y., Okamoto Y., Pratt R.E., Dzau V.J. (2006). Local Renin Angiotensin Expression Regulates Human Mesenchymal Stem Cell Differentiation to Adipocytes. Hypertension.

[52] Park T.S., Zambidis E.T. (2009). A role for the renin-angiotensin system in hematopoiesis. Haematologica.

[53] Khakoo A.Y., Sidman R.L., Pasqualini R., Arap W. (2008). Does the Renin-Angiotensin System Participate in Regulation of Human Vasculogenesis and Angiogenesis?. Cancer Res..

[54] Shen X.Z., Bernstein K.E. (2011). The peptide network regulated by angiotensin converting enzyme (ACE) in hematopoiesis. Cell Cycle.

[55] Vlahakos D.V., Balodimos C., Papachristopoulos V., Vassilakos P., Hinari E., Vlachojannis J.G. (1995). Renin-angiotensin system stimulates erythropoietin secretion in chronic hemodialysis patients. Clin. Nephrol..

[56] Farag E., Sessler D.I., Ebrahim Z., Kurz A., Morgan J., Ahuja S., Maheshwari K., Doyle D.J. (2017). The renin angiotensin system and the brain: New developments. J. Clin. Neurosci..

[57] Al Hmada Y., Brodell R.T., Kharouf N., Flanagan T.W., Alamodi A.A., Hassan S.-Y., Shalaby H., Hassan S.-L., Haikel Y., Megahed M. (2024). Mechanisms of Melanoma Progression and Treatment Resistance: Role of Cancer Stem-like Cells. Cancers.

[58] Cruciat C.-M., Ohkawara B., Acebron S.P., Karaulanov E., Reinhard C., Ingelfinger D., Boutros M., Niehrs C. (2010). Requirement of Prorenin Receptor and Vacuolar H^+^-ATPase–Mediated Acidification for Wnt Signaling. Science.

[59] Zhou L., Li Y., Hao S., Zhou D., Tan R.J., Nie J., Hou F.F., Kahn M., Liu Y. (2015). Multiple Genes of the Renin-Angiotensin System Are Novel Targets of Wnt/β-Catenin Signaling. J. Am. Soc. Nephrol..

[60] Munro M.J., Peng L., Wickremesekera S.K., Tan S.T. (2021). Colon adenocarcinoma-derived cells possessing stem cell function can be modulated using renin-angiotensin system inhibitors. PLoS ONE.

[61] Gajos-Michniewicz A., Czyz M. (2020). WNT Signaling in Melanoma. Int. J. Mol. Sci..

[62] Riddiough G.E., Fifis T., Walsh K.A., Muralidharan V., Christophi C., Tran B.M., Vincan E., Perini M.V. (2021). Captopril, a Renin-Angiotensin System Inhibitor, Attenuates Features of Tumor Invasion and Down-Regulates C-Myc Expression in a Mouse Model of Colorectal Cancer Liver Metastasis. Cancers.

[63] Li J., Fang R., Wu J., Si Y., Bai J., Wang Q. (2022). The NOP14 nucleolar protein suppresses the function and stemness of melanoma stem-like cells through Wnt/beta-catenin signaling inactivation. Bioengineered.

[64] Renziehausen A., Wang H., Rao B., Weir L., Nigro C.L., Lattanzio L., Merlano M., Vega-Rioja A., Fernandez-Carranco M.d.C., Hajji N. (2018). The renin angiotensin system (RAS) mediates bifunctional growth regulation in melanoma and is a novel target for therapeutic intervention. Oncogene.

[65] Vallejo-Ardila D.L., Fifis T., Burrell L.M., Walsh K., Christophi C. (2018). Renin-angiotensin inhibitors reprogram tumor immune microenvironment: A comprehensive view of the influences on anti-tumor immunity. Oncotarget.

[66] Terry S., Savagner P., Ortiz-Cuaran S., Mahjoubi L., Saintigny P., Thiery J., Chouaib S. (2017). New insights into the role of EMT in tumor immune escape. Mol. Oncol..

[67] Zhang H., Yue X., Chen Z., Liu C., Wu W., Zhang N., Liu Z., Yang L., Jiang Q., Cheng Q. (2023). Define cancer-associated fibroblasts (CAFs) in the tumor microenvironment: New opportunities in cancer immunotherapy and advances in clinical trials. Mol. Cancer.

[68] Okwan-Duodu D., Landry J., Shen X.Z., Diaz R. (2013). Angiotensin-converting enzyme and the tumor microenvironment: Mechanisms beyond angiogenesis. Am. J. Physiol. Integr. Comp. Physiol..

[69] Wen S.W., I Ager E., Neo J., Christophi C. (2013). The renin angiotensin system regulates Kupffer cells in colorectal liver metastases. Cancer Biol. Ther..

[70] Simiczyjew A., Dratkiewicz E., Mazurkiewicz J., Ziętek M., Matkowski R., Nowak D. (2020). The Influence of Tumor Microenvironment on Immune Escape of Melanoma. Int. J. Mol. Sci..

[71] Wegman-Ostrosky T., Soto-Reyes E., Vidal-Millán S., Sánchez-Corona J. (2013). The renin-angiotensin system meets the hallmarks of cancer. J. Renin-Angiotensin-Aldosterone Syst..

[72] Tan K.C., Chow W.S., Ai V.H., Metz C., Bucala R., Lam K.S. (2002). Advanced glycation end products and endothelial dysfunction in type 2 diabetes. Diabetes Care.

[73] Liu C., Zhang J.-W., Hu L., Song Y.-C., Zhou L., Fan Y., Zhu H.-Y., Wang Y., Li Q.-P. (2014). Activation of the AT1R/HIF-1α/ACE Axis Mediates Angiotensin II-Induced VEGF Synthesis in Mesenchymal Stem Cells. BioMed Res. Int..

[74] Ma Y., Xia Z., Ye C., Lu C., Zhou S., Pan J., Liu C., Zhang J., Liu T., Hu T. (2019). AGTR1 promotes lymph node metastasis in breast cancer by upregulating CXCR4/SDF-1α and inducing cell migration and invasion. Aging.

[75] Cui T.X., Kryczek I., Zhao L., Zhao E., Kuick R., Roh M.H., Vatan L., Szeliga W., Mao Y., Thomas D.G. (2013). Myeloid-Derived Suppressor Cells Enhance Stemness of Cancer Cells by Inducing MicroRNA101 and Suppressing the Corepressor CtBP2. Immunity.

[76] Radharani N.N.V., Yadav A.S., Nimma R., Kumar T.V.S., Bulbule A., Chanukuppa V., Kumar D., Patnaik S., Rapole S., Kundu G.C. (2022). Tumor-associated macrophage derived IL-6 enriches cancer stem cell population and promotes breast tumor progression via Stat-3 pathway. Cancer Cell Int..

[77] Martínez-Meza S., Díaz J., Sandoval-Bórquez A., Valenzuela-Valderrama M., Díaz-Valdivia N., Rojas-Celis V., Contreras P., Huilcaman R., Ocaranza M.P., Chiong M. (2019). AT2 Receptor Mediated Activation of the Tyrosine Phosphatase PTP1B Blocks Caveolin-1 Enhanced Migration, Invasion and Metastasis of Cancer Cells. Cancers.

[78] Ishikane S., Hosoda H., Nojiri T., Tokudome T., Mizutani T., Miura K., Akitake Y., Kimura T., Imamichi Y., Kawabe S. (2018). Angiotensin II promotes pulmonary metastasis of melanoma through the activation of adhesion molecules in vascular endothelial cells. Biochem. Pharmacol..

[79] Hashemzehi M., Beheshti F., Hassanian S.M., Ferns G.A., Khazaei M., Avan A. (2020). Therapeutic potential of renin angiotensin system inhibitors in cancer cells metastasis. Pathol.-Res. Pr..

[80] Wang J., Nishiyama A., Matsuyama M., Wang Z., Yuan Y. (2020). The (pro)renin receptor: A novel biomarker and potential therapeutic target for various cancers. Cell Commun. Signal..

[81] Huang Y., Noble N., Zhang J., Xu C., Border W. (2007). Renin-stimulated TGF-β1 expression is regulated by a mitogen-activated protein kinase in mesangial cells. Kidney Int..

[82] Cardinale J.P., Sriramula S., Mariappan N., Agarwal D., Francis J. (2012). Angiotensin II–Induced Hypertension Is Modulated by Nuclear Factor-κB in the Paraventricular Nucleus. Hypertension.

[83] Frampton A.E., Sivakumar S. (2022). A New Combination Immunotherapy in Advanced Melanoma. N. Engl. J. Med..

[84] Ribas A., Puzanov I., Dummer R., Schadendorf D., Hamid O., Robert C., Hodi F.S., Schachter J., Pavlick A.C., Lewis K.D. (2015). Pembrolizumab versus investigator-choice chemotherapy for ipilimumab-refractory melanoma (KEYNOTE-002): A randomised, controlled, phase 2 trial. Lancet Oncol..

[85] Larkin J., Chiarion-Sileni V., Gonzalez R., Grob J.-J., Rutkowski P., Lao C.D., Cowey C.L., Schadendorf D., Wagstaff J., Dummer R. (2019). Five-Year Survival with Combined Nivolumab and Ipilimumab in Advanced Melanoma. N. Engl. J. Med..

[86] Patel S.P., Othus M., Chen Y., Wright G.P., Yost K.J., Hyngstrom J.R., Hu-Lieskovan S., Lao C.D., Fecher L.A., Truong T.-G. (2023). Neoadjuvant–Adjuvant or Adjuvant-Only Pembrolizumab in Advanced Melanoma. N. Eng. J. Med..

[87] Tawbi H.A., Schadendorf D., Lipson E.J., Ascierto P.A., Matamala L., Gutiérrez E.C., Rutkowski P., Gogas H.J., Lao C.D., De Menezes J.J. (2022). Relatlimab and Nivolumab versus Nivolumab in Untreated Advanced Melanoma. N. Engl. J. Med..

[88] Nakamura K., Kiniwa Y., Okuyama R. (2021). CCL5 production by fibroblasts through a local renin–angiotensin system in malignant melanoma affects tumor immune responses. J. Cancer Res. Clin. Oncol..

[89] Zhang Z., Wang Z.-X., Chen Y.-X., Wu H.-X., Yin L., Zhao Q., Luo H.-Y., Zeng Z.-L., Qiu M.-Z., Xu R.-H. (2022). Integrated analysis of single-cell and bulk RNA sequencing data reveals a pan-cancer stemness signature predicting immunotherapy response. Genome Med..

[90] Wang B., Zhang W., Zhang G., Kwong L., Lu H., Tan J., Sadek N., Xiao M., Zhang J., Labrie M. (2021). Targeting mTOR signaling overcomes acquired resistance to combined BRAF and MEK inhibition in BRAF-mutant melanoma. Oncogene.

[91] Tomic T., Botton T., Cerezo M., Robert G., Luciano F., Puissant A., Gounon P., Allegra M., Bertolotto C., Bereder J.-M. (2011). Metformin inhibits melanoma development through autophagy and apoptosis mechanisms. Cell Death Dis..

[92] Chaturvedi M.M., Sung B., Yadav V.R., Kannappan R., Aggarwal B.B. (2011). NF-κB addiction and its role in cancer: ‘One size does not fit all’. Oncogene.

[93] Roth I.M., Wickremesekera A.C., Wickremesekera S.K., Davis P.F., Tan S.T. (2019). Therapeutic Targeting of Cancer Stem Cells via Modulation of the Renin-Angiotensin System. Front. Oncol..

[94] Lasithiotakis K.G., Sinnberg T.W., Schittek B., Flaherty K.T., Kulms D., Maczey E., Garbe C., Meier F.E. (2008). Combined Inhibition of MAPK and mTOR Signaling Inhibits Growth, Induces Cell Death, and Abrogates Invasive Growth of Melanoma Cells. J. Investig. Dermatol..

[95] Shao Z., Bao Q., Jiang F., Qian H., Fang Q., Hu X. (2015). VS-5584, a Novel PI3K-mTOR Dual Inhibitor, Inhibits Melanoma Cell Growth In Vitro and In Vivo. PLoS ONE.

[96] Flaherty K.T., Puzanov I., Kim K.B., Ribas A., McArthur G.A., Sosman J.A., O’Dwyer P.J., Lee R.J., Grippo J.F., Nolop K. (2010). Inhibition of Mutated, Activated BRAF in Metastatic Melanoma. N. Engl. J. Med..

[97] Long G.V., Stroyakovskiy D., Gogas H., Levchenko E., de Braud F., Larkin J., Garbe C., Jouary T., Hauschild A., Grob J.J. (2014). Combined BRAF and MEK Inhibition versus BRAF Inhibition Alone in Melanoma. N. Engl. J. Med..

